# A privacy-preserving and computation-efficient federated algorithm for generalized linear mixed models to analyze correlated electronic health records data

**DOI:** 10.1371/journal.pone.0280192

**Published:** 2023-01-17

**Authors:** Zhiyu Yan, Kori S. Zachrison, Lee H. Schwamm, Juan J. Estrada, Rui Duan

**Affiliations:** 1 Department of Neurology, Massachusetts General Hospital, Boston, Massachusetts, United States of America; 2 Department of Emergency Medicine, Massachusetts General Hospital, Boston, Massachusetts, United States of America; 3 Harvard Medical School, Boston, Massachusetts, United States of America; 4 Mass General Brigham, Boston, Massachusetts, United States of America; 5 Department of Biostatistics, Harvard T. H. Chan School of Public Health, Boston, Massachusetts, United States of America; Hanyang University, REPUBLIC OF KOREA

## Abstract

Large collaborative research networks provide opportunities to jointly analyze multicenter electronic health record (EHR) data, which can improve the sample size, diversity of the study population, and generalizability of the results. However, there are challenges to analyzing multicenter EHR data including privacy protection, large-scale computation resource requirements, heterogeneity across sites, and correlated observations. In this paper, we propose a federated algorithm for generalized linear mixed models (Fed-GLMM), which can flexibly model multicenter longitudinal or correlated data while accounting for site-level heterogeneity. Fed-GLMM can be applied to both federated and centralized research networks to enable privacy-preserving data integration and improve computational efficiency. By communicating a limited amount of summary statistics, Fed-GLMM can achieve nearly identical results as the gold-standard method where the GLMM is directly fitted to the pooled dataset. We demonstrate the performance of Fed-GLMM in numerical experiments and an application to longitudinal EHR data from multiple healthcare facilities.

## Introduction

Electronic health records (EHR) data are valuable for generating real-world evidence in biomedical and epidemiological research [1]. With the increasing availability of EHR data among healthcare facilities [2], integrating these data from multiple institutions has great potential for improving statistical power and generalizability of results [3]. Such integration is also particularly valuable—and often necessary—for studying rare conditions and underrepresented subpopulations [4]. As a consequence, an increasing number of clinical research networks have been built domestically and internationally to facilitate multicenter EHR-based studies. For example, the Patient-Centered Outcomes Research Institute has launched PCORnet to support a national research collaborative that empowers large-scale comparative effectiveness research [5]. More recently, large collaborative consortia dedicated to investigating clinical and epidemiological questions about COVID-19 have also been formed [6, 7], as timely observational studies based on large integrated EHR data have been increasingly critical for clinical and health policy decision-making in various areas such as treatment evaluation, diagnostic support and healthcare resource prioritization [8–10].

Despite the importance of multicenter EHR-based studies, challenges exist in terms of how to effectively and efficiently compile and analyze multiple large-scale EHR datasets [11]. Depending on whether the individual-level data are shared, a research network can be categorized into either a *federated* or *centralized* network. A *federated network* keeps patient-level data within each institution, and only allows summary-level statistics to be shared across institutions. Some federated research networks allow automated queries and analysis and share summary statistics through application programming interfaces and cloud computing, which saves human labor from cross-institutional communication but may require additional safeguard against data breaches [12, 13]. Other federated networks rely on manually transferred summary statistics, which has fewer infrastructure requirements, and is considered more reliable for privacy protection. Thus, this practice is widely adopted among international research networks [6, 14, 15]. In federated networks, federated algorithms are needed to conduct joint analyses across multiple datasets without sharing patient-level data. In contrast, *centralized networks* directly pool deidentified patient-level data across institutions and store them in centralized data warehouses [16, 17]. When all data are pooled, fitting a model to a pooled dataset (referred to as the *pooled analysis* hereafter) is feasible but may be subject to challenges from computational complexity and physical memory bottlenecks due to the large size of the pooled dataset. Therefore, in a centralized network, distributed algorithms are also needed to overcome computational challenges [18, 19].

Most of the existing distributed or federated learning algorithms focus on regression models with independent observations, including logistic regression and Cox regression [20–26]. However, EHR data are often longitudinal and correlated, where multiple medical encounters may be associated with the same patient, physician or facility. In EHR-based analyses, methods are needed to account for such multi-level longitudinal and correlated observations. Among many existing methods to address the multi-level correlated data structure, the generalized mixed model (GLMM) is one of the most widely applied methods with great flexibility [27, 28]. To fit a GLMM on federated datasets, one straightforward way is to fit separated models locally across sites and aggregate the local estimates through a meta-analysis [29]. Although meta-analysis is straightforward to implement in practice, it has been shown that its accuracy may be suboptimal, especially when rare conditions are included in the model [23]. A few methods have been developed which consider using site-level random effects to account for heterogeneity across sites. For example, Luo et al. proposed a lossless algorithm for the linear mixed model [30], and a few methods have been proposed for GLMM [31–33]. However, these approaches only consider site-level random effects, which cannot handle repeated and correlated measures within each site. More recently, an algorithm accounting for correlated measures was developed, but only considered a special case for modeling the specificity and sensitivity of diagnostic tests [34]. Methods are needed that allow general and flexible specification of random effects to account for longitudinal or correlated observations at lower levels.

In this paper, we propose an accurate and fast federated algorithm to fit GLMM (Fed-GLMM) with correlated data structures. Our method can be implemented in both federated and centralized networks. Specifically, in a federated setting where the pooled analysis is not feasible, our method provides a solution that only requires a small amount of aggregated data to be shared across sites. Our method requires limited numbers of communications across sites and thus can be applied to both the automated and manual federated settings. In a centralized setting where the pooled analysis is allowed, our method can greatly reduce the computation time and memory cost. In all settings, our method can achieve nearly identical results as the gold-standard pooled analysis estimator, allows flexible specification of random and fixed effects in models, and can account for heterogeneity in the distribution of data across sites. We demonstrate the utility of Fed-GLMM through a real-world EHR data analysis that assesses characteristics associated with virtual versus in-person care utilization during the COVID-19 pandemic, using data from 8 healthcare facilities in the New England area.

## Methods

### GLMM accounting for site-level heterogeneity

Fed-GLMM allows modeling correlated observations from multiple EHRs using the following generic GLMM:

$$g\left(E(y_{ijk}|b_{ijk})\right)=x_{ijk}^{T}\beta +{w_{ijk}^{T}\alpha }_{k}+z_{ijk}^{T}b_{ijk};\;b_{k}∼N(0,B_{k}(\gamma_{k}))$$ (1)

where *g*(⋅) denotes a link function, and *y*_*ijk*_ denotes the outcome variable of the *i*-th visit for the *j*-th patient at the *k*-th site. *x*_*ijk*_, *w*_*ijk*_ and *z*_*ijk*_ denote the corresponding covariates with common fixed effect *β*, site-specific fixed effect *α*_*k*_ and random effect *b*_*ijk*_, respectively, and *b*_*k*_ is a vector containing all the random effects *b*_*ijk*_ for a given *k*. Note that *z*_*ijk*_ is a subset of the union of *x*_*ijk*_ and *w*_*ijk*_. The random effect *b*_*ijk*_ can be flexibly specified to account for different correlation structures. For example, we can include patient-level random effects to account for the correlation among visits of the same patient, and also physician-level random effects to account for the correlation among visits with the same physician.

To account for the heterogeneity across sites, we allow site-specific fixed effect *α*_*k*_ and site-specific variance-covariance structure *B*_*k*_, which is parameterized by *γ*_*k*_. In a homogenous setting, *α*_*k*_ and *B*_*k*_(*γ*_*k*_) can be set equal across sites. Compared with existing work where site-level heterogeneity is adjusted by introducing a random effect [30–33], our method imposes no assumptions on the exchangeability of the site-level effects, which is more robust when the heterogeneity is large, and allows accurate estimation even when the number of sites is very small.

### Fed-GLMM algorithm

**Fig 1:**
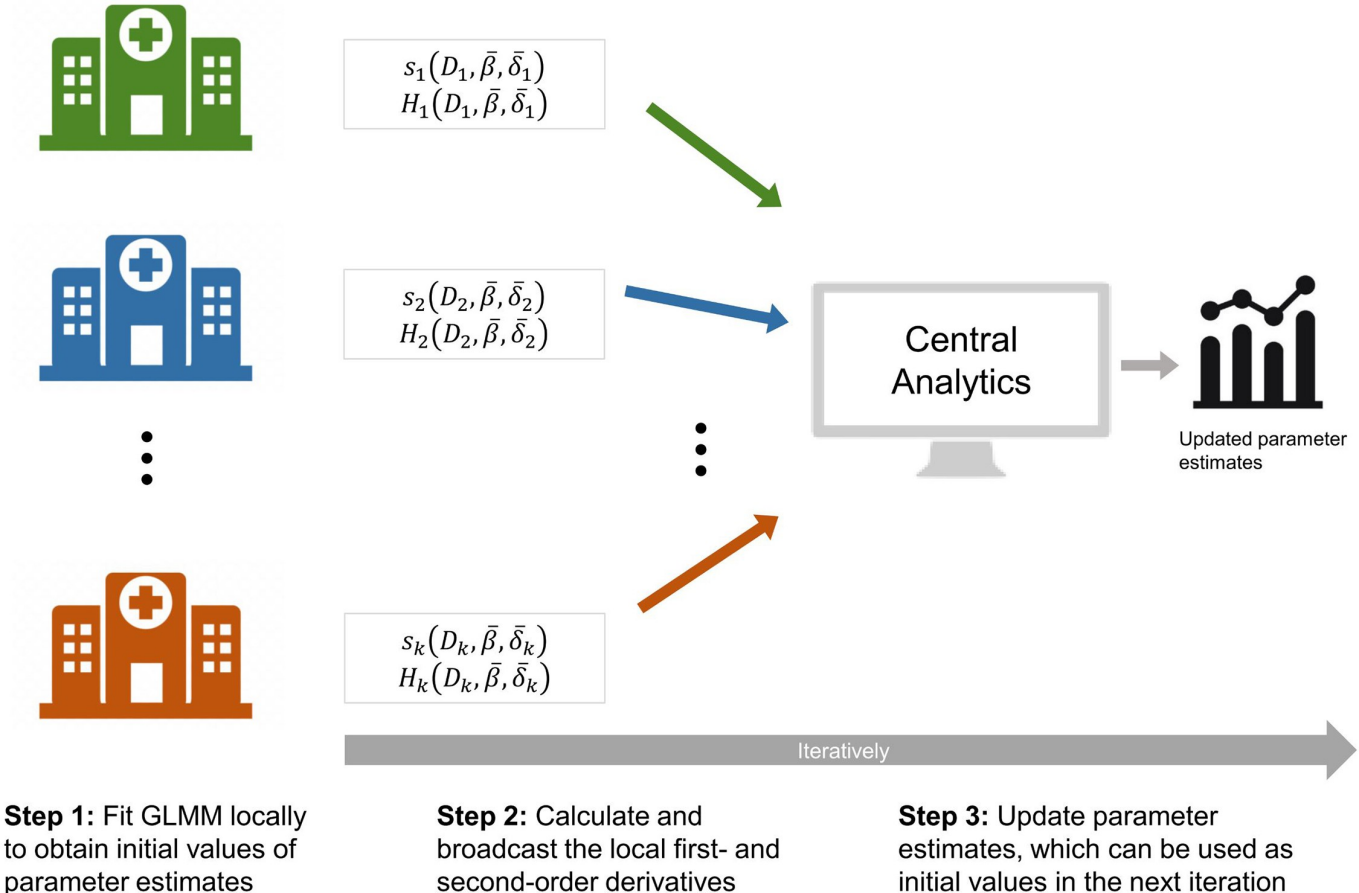
Schematic overview of Fed-GLMM. Fed-GLMM enables the joint implementation of GLMM for EHRs from multiple sites without sharing individual-level data. In step 1, each site fits GLMM locally to obtain the initial parameter estimates. In step 2, each site calculates intermediate summary statistics evaluated at the initial values and broadcasts them to the central analytics. For the *k*-th site, these summary statistics are denoted as *s*_*k*_ and *H*_*k*_, and they are functions of the local data *D*_*k*_, the common parameter value $\bar{\beta }$, and the site-specific parameter value ${\bar{\delta }}_{k}$. The local data *D*_*k*_ is composed of the local design matrix for the common fixed effect *X*_*k*_, the local design matrix for the site-specific fixed effect *W*_*k*_, and the local outcome vector *y*_*k*_. The site-specific parameter value ${\bar{\delta }}_{k}$ is composed of the values of site-specific fixed effect ${\bar{\alpha }}_{k}$ and site-specific variance parameter ${\bar{\gamma }}_{k}$. In step 3, the central analytics combines all the local intermediate results to construct a surrogate global likelihood function that provides updates for parameter estimates. Steps 2–3 can be iteratively performed to keep updating parameter estimates.

We start by introducing an overview of the algorithm. The core concept upon which Fed-GLMM is built is the construction of a quadratic surrogate function using summary statistics collected from each site to approximate the global likelihood function constructed from directly pooling all the data. Fig 1 provides an overview of the Fed-GLMM algorithm. We start with initialization for all the model parameters, denoted by $\bar{\theta }$. Since our model has both common parameters across sites and site-specific parameters, each site is required to fit its own GLMM in the initialization step. The initial values for the site-specific parameters are set to their local estimates (denoted by ${\bar{\delta }}_{k}$ for the *k*-th site), while initial values for the common parameters are updated by a meta-analysis (denoted by $\bar{\beta }$). With more accurate initial values, we can achieve the same level of estimation accuracy with fewer communications across sites. In step 2, each site calculates and broadcasts summary statistics involving less than *p*^2^ numbers (*p* is the number of parameters in the local model). These summary statistics are essentially derivatives of the local likelihood function. In step 3, the summary statistics obtained from step 2 are used to construct a quadratic surrogate function and obtain the parameter updates. When iterative communications are allowed, steps 2–3 can be repeated to further update the parameter values.

To formally introduce the method, suppose we want to integrate data from *K* EHRs stored at *K* different sites. At each site, we fit the GLMM specified earlier (Eq 1). Let *I*_*k*_ denote the index set indicating patients in the *k*-th site. Suppose the *j*-th patient has *n*_*j*_ observations. The log-likelihood function constructed by data from the *k*-th site can then be written as

$$l_{k}(\beta ,\alpha_{k},\gamma_{k})=ln\int \prod_{j\in I_{k}}\prod_{i}^{n_{j}}p_{\alpha_{k},\beta }(y_{ijk}|{x_{ijk},b}_{ijk})p_{\gamma_{k}}(b_{k})db_{k}$$

where $p_{\alpha_{k},\beta }(y_{ijk},x_{ijk}|b_{ijk})$ is the probability density function of *y*_*ijk*_ given *x*_*ijk*_ and the random effect *b*_*ijk*_, and $p_{\gamma_{k}}(b_{k})$ is the distribution of all the random effects *b*_*k*_.

Since the integral above does not have a closed-form solution, the log-likelihood is often approximated by methods such as the penalized quasi-likelihood, the Laplace’s method or Gaussian quadrature [27, 28]. Fed-GLMM applies to any of these integral approximation methods, and here we use Laplace’s method as an example which approximates the log-likelihood by

$${\hat{l}}_{k}(\beta ,\alpha_{k},\gamma_{k})=q({\hat{b}}_{k},\beta ,\alpha_{k},\gamma_{k})-\frac{1}{2}ln|{-\nabla }_{b}^{2}q({\hat{b}}_{i},\beta ,\alpha_{k},\gamma_{k})|$$

where $q(b_{i},\beta ,\alpha_{k},\gamma_{k})=ln(\prod_{j\in I_{k}}\prod_{i}^{n_{j}}p_{\alpha_{k},\beta }(y_{ijk},x_{ijk}|b_{k})p_{\gamma }(b_{k}))$ with *b*_*k*_ evaluated at ${\hat{b}}_{k}$ such that the corresponding first-order derivative $\nabla_{b}q({\hat{b}}_{k},\beta ,\alpha_{k},\gamma_{k})=0$, and $\nabla_{b}^{2}q({\hat{b}}_{k},\beta ,\alpha_{k},\gamma_{k})$ denotes the second-order derivative.

We denote the parameters specific to the *k*-th EHR as *δ*_*k*_ = (*α*_*k*_, *γ*_*k*_), and denote the entire set of parameters as *θ* = (*β*, *δ*_1_, *δ*_2_,…,*δ*_*K*_)^*T*^. Assuming that observations between two different sites are independent, the combined log-likelihood function encompassing all *K* EHRs can be written as

$$\hat{l}(\theta )\equiv \hat{l}(\beta ,\delta_{1}{,\delta }_{2},{\ldots ,\delta }_{K})\equiv \hat{l}(\beta ,\alpha_{1},{\gamma_{1},\alpha }_{2},\gamma_{2},{\ldots ,\alpha }_{K}{,\gamma }_{K})=\sum_{k}^{K}{\hat{l}}_{k}(\beta ,\alpha_{k},\gamma_{k})\equiv \sum_{k}^{K}{\hat{l}}_{k}(\beta ,\delta_{k})$$ (2)

The gold-standard pooled analysis is performed by optimizing the approximated global likelihood function $\hat{l}$ in Eq 2 constructed from data combined from all sites. However, the combined dataset is not always available due to privacy concerns and optimizing the global likelihood function built on a large amount of data can be computationally difficult. Fed-GLMM solves these problems by working with site-specific likelihood function ${\hat{l}}_{k}$ instead of the global likelihood function. More concretely, we propose the following quadratic surrogate function that approximates the global function $\hat{l}(\theta )$ at an initial value $\bar{\theta }={\left(\bar{\beta },{\bar{\delta }}_{1},{\bar{\delta }}_{2},\ldots ,{\bar{\delta }}_{K}\right)}^{T}$:

$$\tilde{l}(\theta ;\bar{\theta })=\hat{l}(\bar{\theta })+{\nabla \hat{l}(\bar{\theta })}^{T}(\theta -\bar{\theta })+\frac{1}{2}{\left(\theta -\bar{\theta }\right)}^{T}\nabla^{2}\hat{l}(\bar{\theta })(\theta -\bar{\theta })$$ (3)

To obtain $\tilde{l}(\theta ;\bar{\theta })$, site *k* needs to share

$$s_{k}=[{\nabla_{\beta }\hat{l}}_{k}{\left(\bar{\mu },{\bar{\delta }}_{k}\right)}^{T},{\nabla_{\delta_{k}}\hat{l}}_{k}{\left(\bar{\mu },{\bar{\delta }}_{k}\right)}^{T}]$$ (4)

$${and\;H}_{k}=\left[\begin{array}{cc} {\nabla_{\beta \beta }\hat{l}}_{k}(\bar{\beta },{\bar{\delta }}_{k}) & {\nabla_{\beta \delta_{k}}\hat{l}}_{k}(\bar{\beta },{\bar{\delta }}_{k})\\ {\nabla_{\delta_{k}\beta }\hat{l}}_{k}(\bar{\beta },{\bar{\delta }}_{k}) & {\nabla_{\delta_{k}\delta_{k}}\hat{l}}_{k}(\bar{\beta },{\bar{\delta }}_{k}) \end{array}\right]$$ (5)

which are summary statistics that can be calculated using the local EHR given an initial value $\bar{\theta }$. When the central analytics receives all the summary statistics, the parameter estimates can be updated through $\tilde{\theta }=argmax_{\theta }\;\tilde{l}(\theta ;\bar{\theta })$. The above procedure can be repeated *T* times when iterative communications are allowed or until a convergence criterion d is reached. We denote the resulting value as *θ*_*Fed*_. The Fed-GLMM algorithm is summarized as Algorithm 1.

Algorithm 1: Fed-GLMM

Step 1 Fit GLMM locally. For *k* in 1,2,…,*K*, do

    • At the *k*-th site, fit a GLMM and obtain initial estimators ${\bar{\delta }}_{k},{\bar{\beta }}_{k}$, as well as the estimated variance of ${\bar{\beta }}_{k}$, denoted by *V*_*k*_

    • Transmit ${\bar{\beta }}_{k}$ and *V*_*k*_ to the central analytics.

Obtain $\bar{\beta }=\frac{\sum V_{k}^{-1}{\bar{\beta }}_{k}}{\sum V_{k}^{-1}}$ and initialize *t* = 0, $\theta^{\left(t\right)}=(\bar{\beta },{\bar{\delta }}_{1},{\bar{\delta }}_{2},\ldots ,{\bar{\delta }}_{K}),$ and Δ = ‖*θ*^(*t*)^‖_2_

While *t*≤*T* or Δ≤d

Step 2 Calculate and broadcast the summary statistics. For *k* in 1,2,…,*K*, do

    • At the *k*-th site, given *θ*^(*t*)^, calculate the first- and second-order derivatives *s*_*k*_ and *H*_*k*_ according to Eqs 4 and 5

    • Transmit the derivatives to the central analytics

Step 3 Update parameter estimates through the central analytics

    • Combine elements of the derivatives from all EHRs to construct the surrogate global likelihood function $\tilde{l}(\theta ;\theta^{\left(t\right)})$ according to Eq 3

    • Obtain $\theta^{\left(t+1\right)}=argmax_{\theta }\;\tilde{l}(\theta ;\theta^{\left(t\right)})$

    • Update Δ = ‖*θ*^(*t*+1)^−*θ*^(*t*)^‖_2_, and *t* = *t*+1

Return *θ*_*Fed*_ = *θ*^*t*^

The algorithm also applies to the homogenous setting where there is no site-specific parameters and our model of interest becomes:

$$g\left(E(y_{ijk}|b_{ijk})\right)=x_{ijk}^{T}\beta +w_{ijk}^{T}\alpha +z_{ijk}^{T}b_{ijk};\;b_{k}∼N(0,B(\gamma ))$$

where the entire set of parameters is redefined as *θ* = (*β*, *α*, *γ*)^*T*^.

The variance of the Fed-GLMM estimator can be calculated directly using the Hessian of the surrogate global likelihood evaluated at *θ*_*Fed*_, denoted as ***H***_*Fed*_. The variance-covariance estimator for *θ*_*Fed*_ can then be obtained through $\hat{Var}(\theta_{Fed})={\left(-H_{Fed}\right)}^{-1}$.

### Simulation study

We use a simulation study to evaluate the accuracy of Fed-GLMM compared to the standard meta-analysis and to evaluate the computation time compared to the gold-standard pooled analysis.

To evaluate the accuracy of Fed-GLMM, we considered a GLMM with a binary outcome (modeled by a logit link function), a binary exposure and three additional covariates (one binary and two continuous variables that follow standard normal and uniform distributions respectively). The model also included a patient-level random intercept and can be expressed as

$$g\left(E(y_{ijk}|b_{jk})\right)=\beta_{0}+x_{1ijk}+0.5*x_{2ijk}+0.5*x_{3ijk}+\alpha_{k}x_{4ijk}+b_{jk};$$

where $x_{1ijk}∼Bernoulli(p_{x}),x_{2ijk}∼Uniform(0,1),x_{3ijk}∼Bernoulli(0.5),\;x_{4ijk}∼N(0,1),\;and\;b_{jk}∼N(0,\gamma_{k}).$

We randomly assigned *k* distinct values ranging from 0 to 1 for the *K* sites as the site-level fixed effect *α*_*k*_, and another *k* distinct values ranging from 0 to 2 as the site-specific variance-component parameter *γ*_*k*_. To study the impact of the prevalence of binary exposure on the model performance, we let *p*_*x*_ vary from 0.01 to 0.5. By choosing different values of *β*_0_, we were also able to allow the prevalence of the binary outcome to vary from 0.01 to 0.5.

In a single simulation replicate, we simulated 8 EHR datasets, each with varying numbers of patients (ranging from 100 to 450) and 5 encounters per patient. We performed the pooled analysis, the meta-analysis and Fed-GLMM respectively using the same set of datasets. We evaluated the accuracy of Fed-GLMM versus meta-analysis by the relative bias for estimating the coefficient of the binary exposure *x*_1_, calculated as the following:

$$Relative\;Bias=|\frac{\mathrm{Fed}‐\mathrm{GLMM}\;or\;\mathrm{Meta}‐\mathrm{analysis}\;Estimate-\mathrm{Pooled}\;\mathrm{Analysis}\;Estimate}{\mathrm{Pooled}\;\mathrm{Analysis}\;Estimate}|$$

To evaluate the computational efficiency of Fed-GLMM, we considered the same GLMM specifications and variable distributions as in the previous simulation except that the site-specific parameters *α*_*k*_ and *γ*_*k*_ are fixed at 0.5 and 1 respectively for all *k*. In a single simulation replicate, we generated a single dataset with 5,000 patients and 5 encounters per patient (25,000 encounters in total). The prevalence of the binary exposure was set as 0.05 and the prevalence of binary outcome was set as 0.25. We applied Fed-GLMM by randomly splitting the dataset into subsets. We investigated the computation time with the number of subsets ranging from 5 to 100. We generated 50 simulation replicates for each setting, and for each simulation replicate, we also performed the pooled analysis and the meta-analysis. We evaluated the computational efficiency of Fed-GLMM and meta-analysis using the ratio of their computation time to that of the pooled analysis, calculated as the following:

$$Relative\;Computation\;Time=\frac{\mathrm{Fed}‐\mathrm{GLMM}\;or\;\mathrm{Meta}‐\mathrm{analysis}\;Computation\;Time}{\mathrm{pooled}\;\mathrm{Analysis}\;Computation\;Time}$$

### An application of Fed-GLMM to real-world EHR data

#### Data source

We applied Fed-GLMM to assess characteristics associated with a dataset of real-world virtual versus in-person care utilization during the COVID-19 pandemic. The dataset was curated from the data warehouse of a large New England healthcare system. For a demonstration of Fed-GLMM, we included all ambulatory visits with physicians conducted at 8 acute care hospital facilities during a one-year period from 10/1/2020 through 9/30/2021. We extracted patient characteristics and demographics, physician primary specialty, and whether the visit was conducted in person or virtually through associated modifier codes. This EHR study was approved by our Institutional Review Board as a medical record review that did not require patient consent.

#### Model specifications

We considered a GLMM with a binary outcome indicating whether a care encounter was conducted virtually (coded as 1) or in person (coded as 0). The covariates in the model included patient age, gender, race/ethnicity (Hispanic, non-Hispanic white, non-Hispanic black, non-Hispanic Asian and other non-Hispanic race/ethnicity), English proficiency (whether the patient indicated English as the preferred language), the digital patient portal status (whether the portal was activated or not for the care encounter as a proxy for “digital literacy”), insurance status (whether the visit was billed to the Medicaid as a proxy for social determinants of health), visit type (whether the visit was completed in a primary care, behavioral health or specialty department), and an indicator for whether the care happened on or after 5/29/2021—the ending of social restriction in Massachusetts (to approximate the beginning of “social normalization” during the COVID-19 pandemic in the New England area). The fixed effect intercept was modeled to be site-specific to accommodate the varying prevalence of virtual care across facilities. We included a physician-level random intercept and a patient-level random intercept to account for the correlations across encounters at different levels. We set the variance components parameterizing the random effects to be homogeneous across sites due to the supporting evidence from site-specific models and the need for improving statistical efficiency.

#### Centralized setting

We demonstrate the computation time benefit of Fed-GLMM compared to the pooled analysis in a centralized setting. We fit the same GLMM using the EHR data from the facility with the highest visit volume. With Fed-GLMM, the single EHR was split into 10 smaller subsets to be computed in parallel. Our analysis was performed with R 4.0.2 on a Linux cluster with up to 512GB RAM per node at 1600MHz. Different from the simulation study where we had only the patient-level random effects, physician-level random effects were also included in our real-world data analysis. This added difficulties in terms of dividing data into subsets. When there are only patient-level random effects in the model, we can randomly split the patients into subsets. However, when there are physician-level random effects, a random split of patients cannot guarantee two observations associated with the same physician are in the same subset, and a random split of physicians cannot guarantee two observations associated with the same patient are in the same subset. To keep correlated observations in the same subset as much as possible, we propose a clustering-based algorithm such that two physicians are more likely to be assigned to the same subsets if they share more patients. In this way, we can best preserve the correlation structure of the data, and therefore achieve better accuracy. The detailed procedures of the cluster-splitting algorithm are demonstrated in S1 Table. To evaluate and exemplify the algorithm, we also repeatedly apply it to small subsets of our real-world EHR data at a facility with a large number of records and compared the resulting Fed-GLMM estimates with the estimates obtained using randomly splitting the data, both relative to the gold-standard pooled analysis results which are obtainable for subset data.

#### Federated setting

To demonstrate the use case of Fed-GLMM in a federated setting, we applied it to EHRs from all 8 facilities to fit the GLMM with facility-specific fixed intercepts that account for heterogeneity across facilities. EHRs from the two largest facilities were each split into 10 subsets to reduce computation time, while other smaller EHRs were not split.

## Results

### Simulation study

#### Fed-GLMM demonstrated improved accuracy compared to meta-analysis in a federated setting

**Fig 2:**
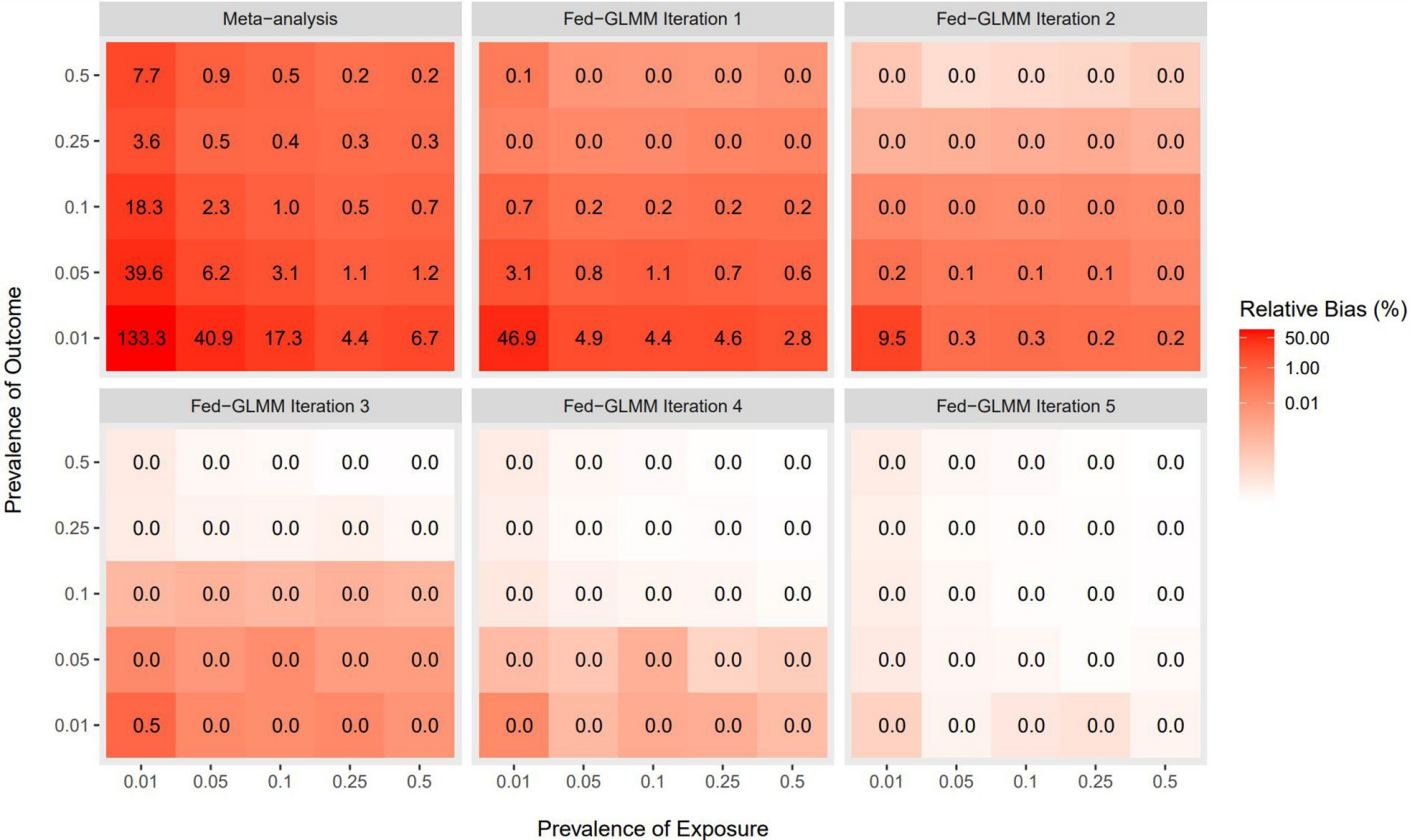
Accuracy of Fed-GLMM and meta-analysis estimates relative to the gold-standard pooled analysis. We compared the accuracy of Fed-GLMM with the standard meta-analysis by calculating the median absolute relative difference compared to the gold-standard pooled estimate of the coefficient of a binary exposure variable. The underlying model has a binary outcome, a binary exposure, three more covariates with 8 site-specific fixed effect coefficients for the normally distributed covariate and a patient-level random intercept. The model also includes 8 site-specific parameters for variance components. We considered 25 combinations of outcome and exposure prevalence to assess the model accuracy with 100 simulation replicates per combination. Fed-GLMM demonstrated reduced relative bias after 1–2 iterations compared with the meta-analysis, which was highly biased in the presence of rare events.

From Fig 2 (upper left panel), the relative bias of the meta-analysis estimator for the exposure coefficient was more severe with rare outcomes or exposures. In contrast, Fed-GLMM converged to the values nearly identical to the pooled analysis estimates within 5 iterations in all prevalence settings. Additionally, in most non-rare event settings, Fed-GLMM achieved considerable improvement over the meta-analysis within 1–2 iterations. Compared with the meta-analysis, Fed-GLMM also demonstrated small variability in bias from the pooled analysis estimates across the simulation replicates, as shown in S1 Fig.

#### Fed-GLMM demonstrated improved computational efficiency compared to the pooled analysis in a centralized setting

**Fig 3:**
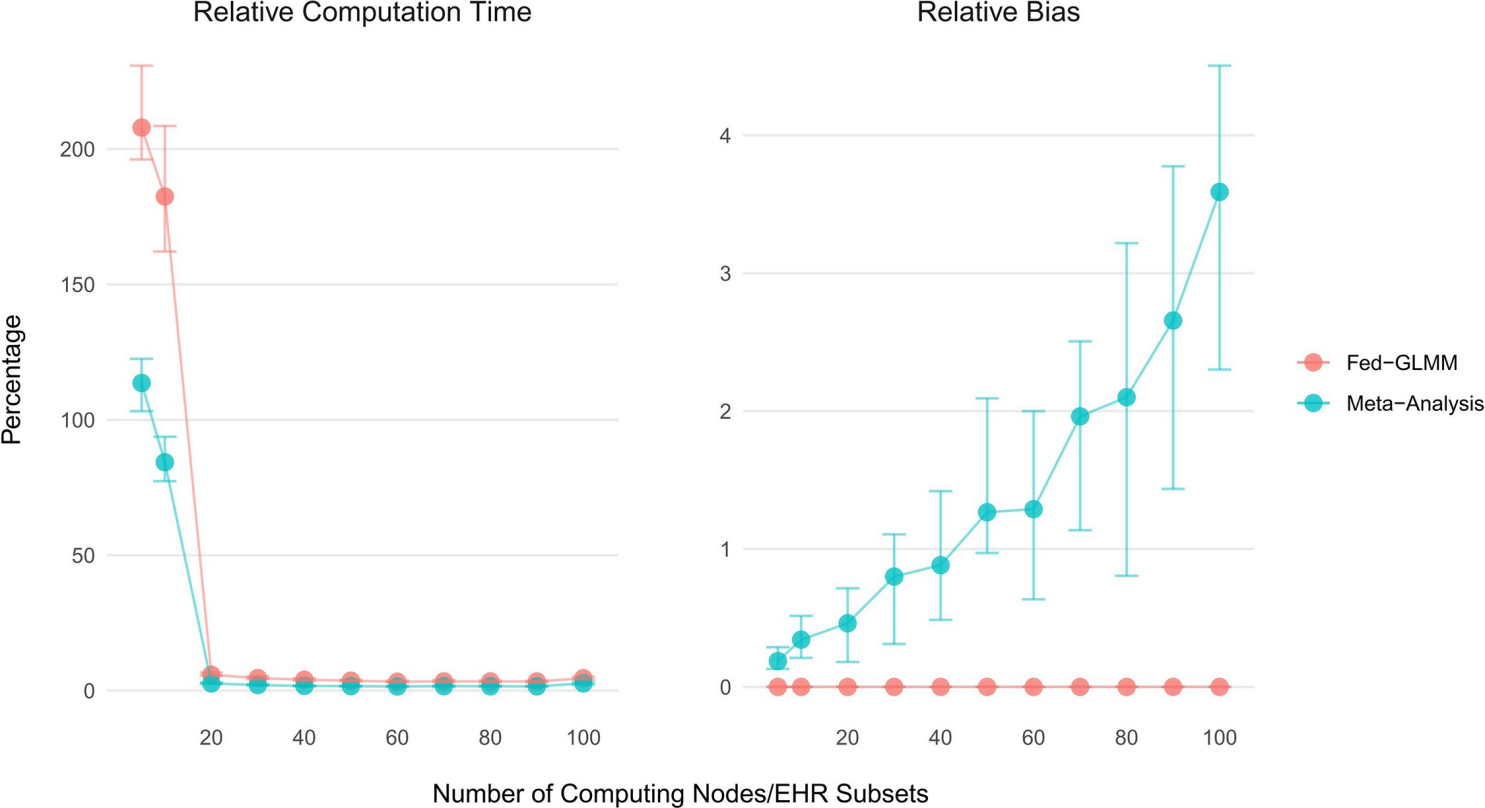
Comparison of computation time and estimate accuracy for Fed-GLMM and meta-analysis relative to gold-standard pooled analysis with increasing computing nodes/EHR subsets. We compared Fed-GLMM with meta-analysis using the ratio (in percentage) of computation time over the pooled analysis. For each simulation replicate, we generated one single centralized EHR. The underlying model has a binary outcome, a binary exposure, three more covariates and a patient-level random intercept. We considered dividing the centralized EHR data into varying numbers of subsets to be computed in parallel. Both Fed-GLMM and the meta-analysis spent less than 5% of the computation time required by the pooled analysis with the number of computing nodes greater than 20. However, the meta-analysis had increased relative bias for the exposure coefficient when the number of subsets increased, while Fed-GLMM retained its accuracy relative to the pooled analysis. The points and bars represent median and interquartile range of computation time and relative bias in percentage respectively.

We divided a pooled dataset into a different number of subsets and Fed-GLMM is applied using multiple computing nodes in parallel. Fig 3 shows that Fed-GLMM spent than 5% of the computation time required by the pooled analysis when the number of computing nodes exceeds 20, and the time can be further reduced with more computing nodes. The meta-analysis can also provide a similar time reduction effect through parallel computing. However, with more computing nodes, the meta-analysis resulted in increasing relative bias, while Fed-GLMM retained its accuracy relative to the pooled analysis.

### Real-world data analysis

We identified 3,165,913 outpatient encounters between 10/1/2020 and 9/30/2021 from the 8 acute care hospital facilities in the New England area. We performed a complete-case analysis where all observations with missing values (215,329 visits) were excluded from the final analytical sample. The descriptive statistics for all covariates are shown in S2 Table. Before performing Fed-GLMM, we estimated variance-component parameters locally and separately for each site. As shown in S4 Table, those estimates are similar among the sites for both patient- and physician-level random effects, supporting our model specification of equal variance-components parameters.

**Fig 4:**
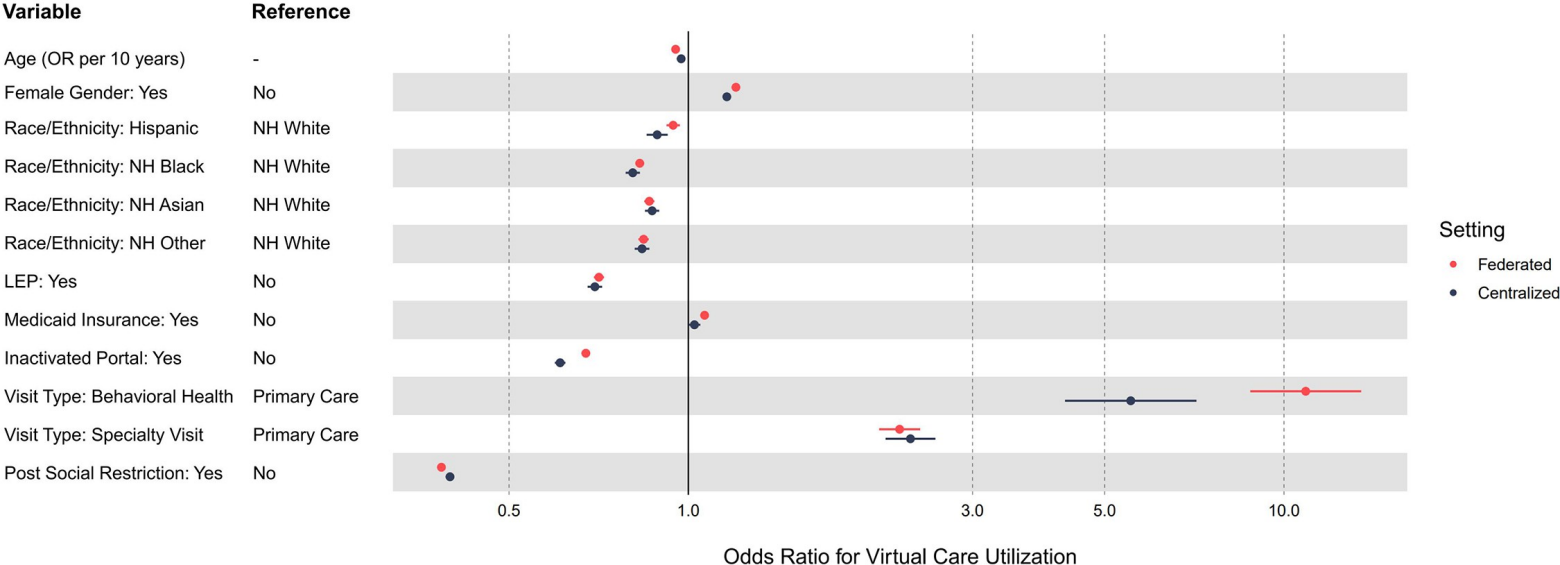
Adjusted odds ratios of virtual vs. in-person visit by patient and visit characteristics. Using the forest plot, we visualized the adjusted odds ratios obtained through Fed-GMM for both all facilities (federated setting to demonstrate privacy preservation) and single facility (centralized setting to demonstrate computation improvement). The points and bars represent the point estimates and 95% confidence intervals, respectively. Abbreviations: OR—Odds Ratio; NH—Non-Hispanic; LEP—Limited English Proficiency.

The results of Fed-GLMM for the federated (all 8 facilities) and centralized (the facility with the highest visit volume only) settings are summarized in Fig 4. The detailed numeric outputs are displayed in S3 Table. For the centralized setting, the entire process took around 1.4 hours, while the analysis would be otherwise infeasible within a similar timeframe if all data were fit in a single GLMM process.

To address the computational concern, analyses in both federated and centralized settings involve splitting data at facilities with a large number of records. We evaluated the performance of the clustering-based splitting method designed to address the issue of crossed patient- and physician-level random effects as described in Methods. As demonstrated in S2 Fig, for all coefficients of interest, clustering-based splitting resulted in negligible bias compared with the random splitting strategy. This also demonstrated the good performance of Fed-GLMM in the real-world data scenario given that an appropriate splitting strategy is applied.

In both federated and centralized settings, the characteristics associated with lower odds of conducting a virtual visit (i.e., higher odds of conducting an in-person visit) are increasing age, Hispanic, non-Hispanic Black, non-Hispanic Asian or other non-Hispanic relative to non-Hispanic White race/ethnicity, limited English proficiency, and inactive patient portal. Compared with primary care, behavioral health and specialty visits were more likely to be conducted virtually. Visits of female patients, as well as visits billed to Medicaid were also more likely to be conducted virtually. Except for the behavior health specialty, the parameter estimates for the federated and centralized settings are very close to each other, demonstrating the potential dominant effects of the site with the largest proportion of data on the results based on all sites.

## Discussion

In light of the increasing need for multicenter collaborative research utilizing EHR data and the potential challenges in data sharing and large-scale computation, we proposed the Fed-GLMM algorithm to model correlated EHR data that allows privacy-preserving integration of datasets from multiple healthcare systems. Our method enables fitting GLMM with much less computation time and memory cost in both federated and centralized networks, and thus can also be applied to EHR from a single site. Our simulation study has demonstrated that Fed-GLMM achieves nearly identical results to the pooled analysis with reduced computation time over a broad spectrum of settings. Our real-world data analysis demonstrated the feasibility of applying Fed-GLMM to single-site and multicenter EHR-based studies to fit a model with millions of observations.

In contrast to a meta-analysis, our method is not based on constructing a weighted average of local estimators obtained from each site. When studying rare events, the local estimators would be biased due to the limited number of cases, which can lead to biases in the meta-analysis. Our method is more robust to such biases as we directly aggregate the first- and second- order derivatives, which are not sensitive to the rareness of the event. The contribution of each study in Fed-GLMM is captured by the shared derivatives, which cannot be summarized to a single weight as in a univariate meta-analysis. Nevertheless, compared to existing work of federated and distributed algorithms, the most important contribution of Fed-GLMM is that it allows the modeling of longitudinal and correlated data within each institution and can accommodate all GLMM specifications, including crossed or nested random effects. However, when performing Fed-GLMM to improve computational efficiency through splitting large-scale data in a centralized setting, one needs to be mindful of the data-splitting strategy. For nested random effects, splitting the data by the highest-level factors will allow Fed-GLMM estimates to converge to the gold-standard pooled analysis results. For crossed random effects, we recommend splitting the data such that the correlated observations are allocated to the same subsets as much as possible as shown in S2 Fig. This makes the Fed-GLMM estimates close (though not identical) to the pooled analysis estimates.

As demonstrated in our simulation and real-world data analyses, iterative communication among the central analytics and individual sites is not required. In most cases, only one round of parameter updating provides negligible bias. Thus, in federated research networks that rely on manual data transferring, our method with one round of iteration is preferred to reduce the communication cost. However, when multiple rounds of communication are feasible, with an increasing number of iterations, our method will eventually converge to the pooled analysis. When studying rare conditions, extra iterations help correct the bias, so a balance needs to be reached between the communication cost and estimation accuracy. In addition, the sharing of first- and second-order derivatives is common among federated algorithms but may still entail a risk of identifiability for small datasets with rare events. Nevertheless, this risk is limited in that the transmission of summary-level statistics is typically regulated and protected by the data-sharing protocols of collaborative research networks. Methods such as differential privacy and data encryption techniques can be combined with Fed-GLMM to improve privacy protection. While we have demonstrated Fed-GLMM for analyzing EHR data to assess virtual care utilization, our method can be used to address correlated structures in many types of real-world datasets. Future steps include paring Fed-GLMM with high-dimensional data analysis methods to model genetic datasets, as well as the combination of Fed-GLMM and vertical data integration techniques to integrate granular clinical and health service information from longitudinal administrative claims and survey databases [35].

## Supporting information

S1 Fig Absolute relative bias of Fed-GLMM and meta-analysis estimates relative to gold-standard pooled analysis across simulation replicates. We compared the accuracy of Fed-GLMM with the standard meta-analysis by calculating the absolute relative difference from the gold-standard pooled analysis for estimating the coefficient of a binary exposure variable. The underlying model has a binary outcome, a binary exposure, three more covariates with 8 site-specific fixed effect coefficients for the normally distributed covariate and a patient-level random intercept. The model also includes 8 site-specific parameters for variance components. We considered 25 combinations of outcome and exposure prevalence each with 100 simulation replicates to assess the model accuracy. Fed-GLMM achieved almost identical results as the pooled analysis for all simulation replicates after 1–2 iterations, while the meta-analysis demonstrated greater bias and variance relative to the pooled analysis across all simulation replicates and prevalence settings. Abbreviations: EY—Prevalence of Outcome; EX—Prevalence of Exposure.
(JPG)

S2 Fig Comparison of Fed-GLMM accuracy relative to pooled analysis between different data splitting strategies. Using randomly extracted small sub-datasets (n = 100,000) from the EHR of a single facility, we compare the accuracy of Fed-GLMM with different data splitting strategies. Two splitting strategies were attempted and compared: random splitting and our proposed clustering-based splitting introduced in S**1 Table**. A sub-dataset was split into 5 subsets by both strategies. The absolute relative bias was calculated as the difference between the corresponding Fed-GLMM estimates and those given by the pooled analysis in absolute percentage. A total of 50 randomly extracted sub-datasets were used in the evaluation. For all coefficients of interests, clustering-based splitting resulted in negligible bias compared with the random splitting strategy. Abbreviations: NH—Non-Hispanic; LEP—Limited English Proficiency.
(JPG)

S1 Table Clustering-based splitting strategy. This strategy aims to cluster physicians who shared patients together by examining physicians’ patient-sharing network. With the clustering-based splitting strategy, two physicians are more likely to be assigned to the same subsets if they share more patients so that the patients’ visits linked to multiple physicians are likely to be included in the same data subsets.
(DOCX)

S2 Table Descriptive statistics for the virtual care utilization analysis. We identified all outpatient encounters spanning 10/1/2020 through 9/30/2021 from the 8 acute care hospital facilities in the New England area, which we deidentified and denoted as Facilities 1 through 8. We described all the variables in the dataset to be used in the Fed-GLMM analysis, as well as the overall observation distribution across facilities.
(DOCX)

S3 Table Parameter estimates generated by Fed-GLMM for the virtual care utilization analysis. We displayed the adjusted odds ratios with 95% confidence intervals obtained through Fed-GLMM for both single EHR from Facility 4 (centralized setting to demonstrate computation improvement) and all facilities (federated setting to demonstrate privacy preservation). We adopted a complete-case analysis where 6.8% of the observations with missing values were removed. Abbreviation: Ref—Reference Group.
(DOCX)

S4 Table Local variance parameter estimates at each facility. We demonstrated variance-component parameter estimates obtained separately for each site to evaluate our assumption of equal variance-component parameters across sites. For sites with a relatively small number of records, the estimates were obtained by fitting the GLMM locally. For sites with a large number of records, the estimates were obtained by a meta-analysis aggregating the GLMM estimates from split data batches. The variance- component parameter estimates for both patient- and physician-level random effects are similar across the sites, supporting our model specification with homogenous variance-component parameters.
(DOCX)
