# Supplementary figures and images for: A privacy-preserving and computation-efficient federated algorithm for generalized linear mixed models to analyze correlated electronic health records data
Source: PLoS One. 2023 Jan 17;18(1):e0280192. doi: 10.1371/journal.pone.0280192 (PMC9844867; doi:10.1371/journal.pone.0280192)

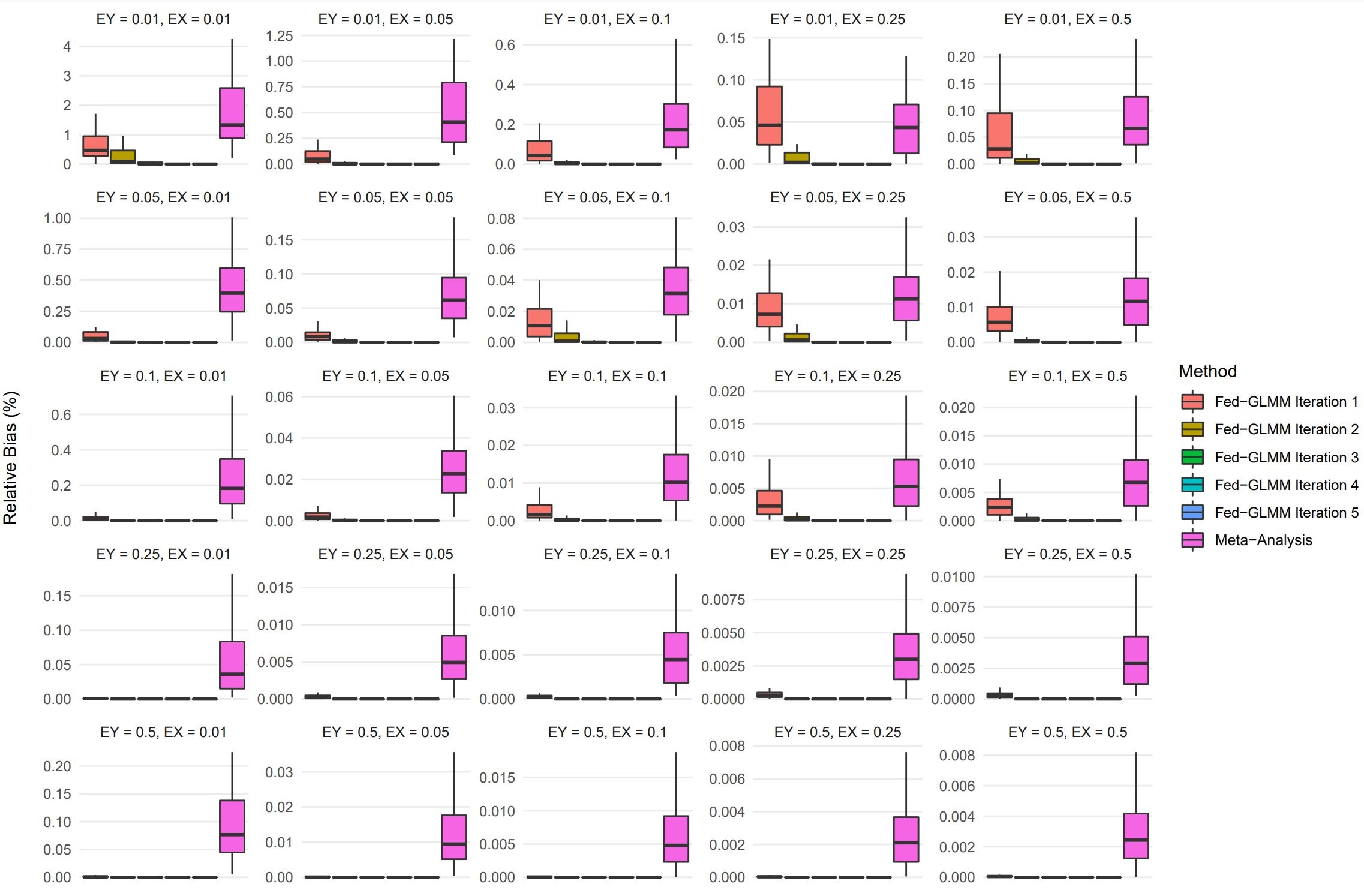

Supplement: S1 Fig — We compared the accuracy of Fed-GLMM with the standard meta-analysis by calculating the absolute relative difference from the gold-standard pooled analysis for estimating the coefficient of a binary exposure variable. The underlying model has a binary outcome, a binary exposure, three more covariates with 8 site-specific fixed effect coefficients for the normally distributed covariate and a patient-level random intercept. The model also includes 8 site-specific parameters for variance components. We considered 25 combinations of outcome and exposure prevalence each with 100 simulation replicates to assess the model accuracy. Fed-GLMM achieved almost identical results as the pooled analysis for all simulation replicates after 1–2 iterations, while the meta-analysis demonstrated greater bias and variance relative to the pooled analysis across all simulation replicates and prevalence settings. Abbreviations: EY—Prevalence of Outcome; EX—Prevalence of Exposure. (JPG) [file pone.0280192.s001.JPG]

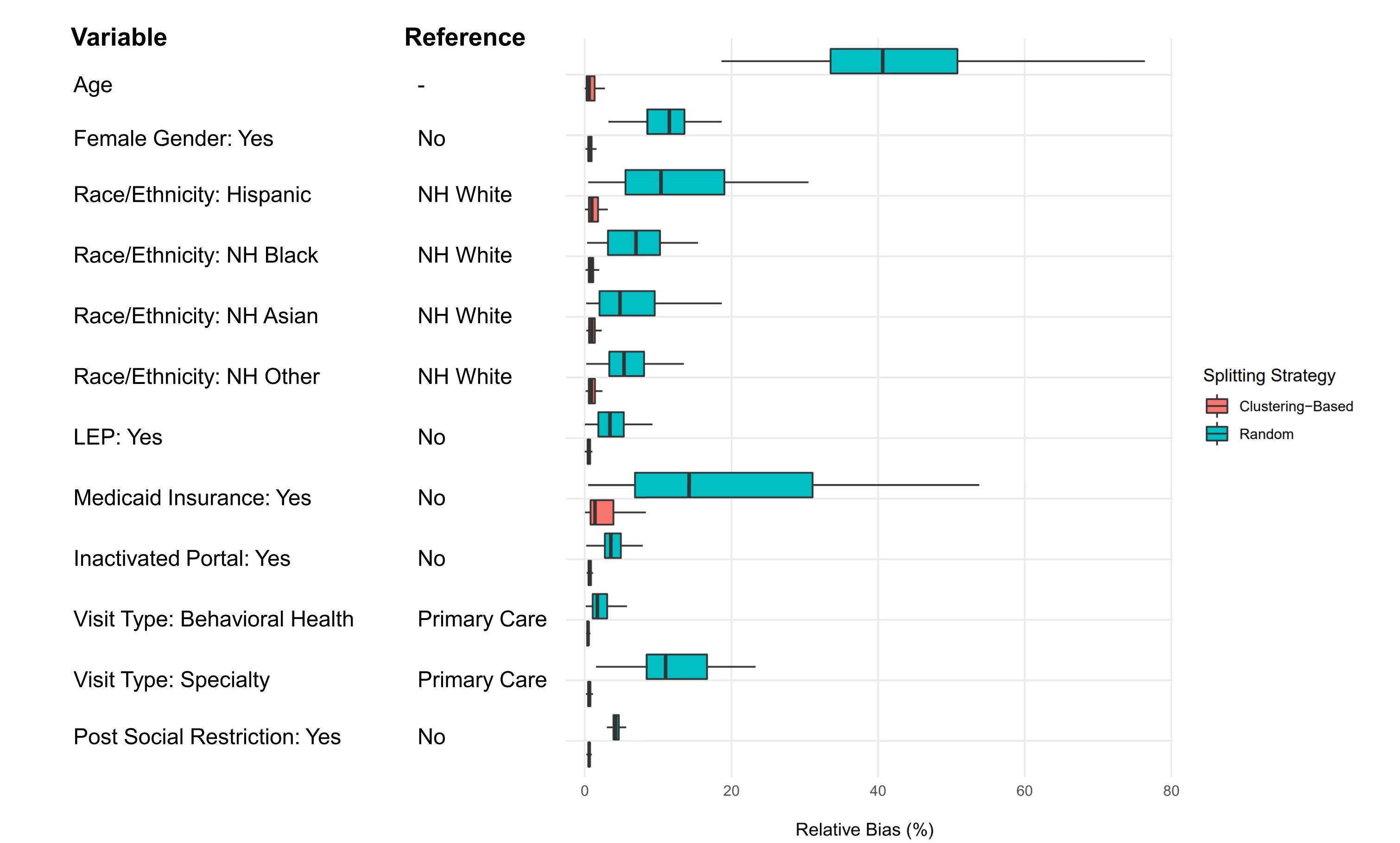

Supplement: S2 Fig — Using randomly extracted small sub-datasets (n = 100,000) from the EHR of a single facility, we compare the accuracy of Fed-GLMM with different data splitting strategies. Two splitting strategies were attempted and compared: random splitting and our proposed clustering-based splitting introduced in S1 Table. A sub-dataset was split into 5 subsets by both strategies. The absolute relative bias was calculated as the difference between the corresponding Fed-GLMM estimates and those given by the pooled analysis in absolute percentage. A total of 50 randomly extracted sub-datasets were used in the evaluation. For all coefficients of interests, clustering-based splitting resulted in negligible bias compared with the random splitting strategy. Abbreviations: NH—Non-Hispanic; LEP—Limited English Proficiency. (JPG) [file pone.0280192.s002.JPG]
