# Supplementary material for: A privacy-preserving and computation-efficient federated algorithm for generalized linear mixed models to analyze correlated electronic health records data
Source: PLoS One. 2023 Jan 17;18(1):e0280192. doi: 10.1371/journal.pone.0280192 (PMC9844867; doi:10.1371/journal.pone.0280192)
Supplement: S1 Table — This strategy aims to cluster physicians who shared patients together by examining physicians’ patient-sharing network. With the clustering-based splitting strategy, two physicians are more likely to be assigned to the same subsets if they share more patients so that the patients’ visits linked to multiple physicians are likely to be included in the same data subsets. (DOCX) [file pone.0280192.s003.docx]

| Clustering-based Data-Splitting Strategy for Crossed Patient and Physician Random Effects |
| --- |
| Step 1 Obtain the edge list from the physicians’ patient-sharing network   - Step 1.1 Create a binary contingency table for patients and physicians from the large EHR dataset, stored as matrix $P$, where each cell indicates whether at least one visit happened involving the corresponding patient and physician - Step 1.2 Construct physicians’ patient-sharing network from the weighted adjacency matrix $P^{T}P$ - Step 1.3 Extract the weighted edge list from the network, where the weight of an edge indicates the number of patients shared by the two physicians on the edge |
| Step 2 Cluster physicians according to edge weights   - Step 2.1 Sort the edge list by edge weights in descending order - Step 2.2 To split the large-scale EHR data into $A$ subsets, choose $A-1$ cut points for the edge weights to cut the full edge list into $A$ sub-edge list in sequence such that the $A$ subsets obtained in Step 3 have approximately uniform sizes. - Step 2.3 Allocate all physicians involved in the first sub-edge list (the one with the highest weights) into the first cluster, and then allocate all physicians in the second sub-edge list into the second cluster, excluding those already classified into the first cluster; repeat the allocation steps through all sub-edge lists to obtain $A$ physician clusters. |
| Step 3 Split the large-scale EHR into $A$ subsets according to $A$ physician clusters. |
