# Supplementary material for: A privacy-preserving and computation-efficient federated algorithm for generalized linear mixed models to analyze correlated electronic health records data
Source: PLoS One. 2023 Jan 17;18(1):e0280192. doi: 10.1371/journal.pone.0280192 (PMC9844867; doi:10.1371/journal.pone.0280192)
Supplement: S2 Table — We identified all outpatient encounters spanning 10/1/2020 through 9/30/2021 from the 8 acute care hospital facilities in the New England area, which we deidentified and denoted as Facilities 1 through 8. We described all the variables in the dataset to be used in the Fed-GLMM analysis, as well as the overall observation distribution across facilities. (DOCX) [file pone.0280192.s004.docx]

| ­**Variable** | **In-person (N=2,208,847)** | **Virtual (N=957,066)** | **Overall (N=3,165,913)** |
| --- | --- | --- | --- |
| **Age** |  |  |  |
| Median (Q1-Q3) | 58.0 (38.0-71.0) | 55.0 (35.0-69.0) | 57.0 (37.0-70.0) |
| Missing | 0 (0%) | 7 (0.0%) | 7 (0.0%) |
| **Gender** |  |  |  |
| Female | 1,322,426 (59.9%) | 595,298 (62.2%) | 1,917,724 (60.6%) |
| Male | 886,371 (40.1%) | 361,747 (37.8%) | 1,248,118 (39.4%) |
| Missing | 50 (0.0%) | 21 (0.0%) | 71 (0.0%) |
| **Race and Ethnicity** |  |  |  |
| Non-Hispanic White | 1,758,356 (79.6%) | 754,704 (78.9%) | 2,513,060 (79.4%) |
| Hispanic | 43,069 (1.9%) | 27,677 (2.9%) | 70,746 (2.2%) |
| Non-Hispanic Black | 119,142 (5.4%) | 54,833 (5.7%) | 173,975 (5.5%) |
| Non-Hispanic Asian | 95,088 (4.3%) | 40,262 (4.2%) | 135,350 (4.3%) |
| Non-Hispanic Other | 97,948 (4.4%) | 44,858 (4.7%) | 142,806 (4.5%) |
| Missing | 95,244 (4.3%) | 34,732 (3.6%) | 129,976 (4.1%) |
| **Language** |  |  |  |
| English | 2,056,430 (93.1%) | 897,908 (93.8%) | 2,954,338 (93.3%) |
| Not English | 149,775 (6.8%) | 58,679 (6.1%) | 208,454 (6.6%) |
| Missing | 2,642 (0.1%) | 479 (0.1%) | 3,121 (0.1%) |
| **Medicaid Insurance** |  |  |  |
| Yes | 193,083 (8.7%) | 102,712 (10.7%) | 295,795 (9.3%) |
| No | 1,981,861 (89.7%) | 840,850 (87.9%) | 2,822,711 (89.2%) |
| Missing | 33,903 (1.5%) | 13,504 (1.4%) | 47,407 (1.5%) |
| **Patient Portal Activation Status** |  |  |  |
| Activated | 1,790,533 (81.1%) | 844,577 (88.2%) | 2,635,110 (83.2%) |
| Inactivated | 389,216 (17.6%) | 106,586 (11.1%) | 495,802 (15.7%) |
| Missing | 29,098 (1.3%) | 5,903 (0.6%) | 35,001 (1.1%) |
| **Visit Type** |  |  |  |
| Primary Care | 706,394 (32.0%) | 245,510 (25.7%) | 951,904 (30.1%) |
| Behavioral Health | 7,256 (0.3%) | 117,594 (12.3%) | 124,850 (3.9%) |
| Specialty | 1,493,186 (67.6%) | 582,175 (60.8%) | 2,075,361 (65.6%) |
| Missing | 2,011 (0.1%) | 11,787 (1.2%) | 13,798 (0.4%) |
| **Post Social Restriction** |  |  |  |
| Yes | 796,219 (36.0%) | 235,854 (24.6%) | 1,032,073 (32.6%) |
| No | 1,412,628 (64.0%) | 721,212 (75.4%) | 2,133,840 (67.4%) |
| **Encounter Count (Patient Count) for Each Facility** |  |  |  |
| Facility 1 | 89,778 (48,161) | 51,411 (29,485) | 141,189 (63,898) |
| Facility 2 | 681,638 (275,880) | 260,212 (133,705) | 941,850 (320,654) |
| Facility 3 | 126,695 (48,105) | 27,486 (14,079) | 154,181 (50,936) |
| Facility 4 | 808,473 (313,386) | 479,124 (199,529) | 1,287,597 (390,110) |
| Facility 5 | 23,731 (10,544) | 5,774 (3,092) | 29,505 (11,226) |
| Facility 6 | 182,776 (87,743) | 65,661 (37,797) | 248,437 (98,764) |
| Facility 7 | 123,455 (66,306) | 51,686 (29,574) | 175,141 (77,424) |
| Facility 8 | 172,301 (65,707) | 15,712 (10,407) | 188,013 (67,746) |
