# Supplementary material for: A privacy-preserving and computation-efficient federated algorithm for generalized linear mixed models to analyze correlated electronic health records data
Source: PLoS One. 2023 Jan 17;18(1):e0280192. doi: 10.1371/journal.pone.0280192 (PMC9844867; doi:10.1371/journal.pone.0280192)
Supplement: S3 Table — We displayed the adjusted odds ratios with 95% confidence intervals obtained through Fed-GLMM for both single EHR from Facility 4 (centralized setting to demonstrate computation improvement) and all facilities (federated setting to demonstrate privacy preservation). We adopted a complete-case analysis where 6.8% of the observations with missing values were removed. Abbreviation: Ref—Reference Group. (DOCX) [file pone.0280192.s005.docx]

| **Variable** | **Federated Setting**  **(N = 2,950,584)** | **Centralized Setting**  **(N = 1,194,009)** |
| --- | --- | --- |
| **Age (Odds Ratio per 10 year)** | 0.95 (0.95 - 0.95) | 0.97 (0.97 - 0.98) |
| **Female Gender** | 1.20 (1.19 - 1.21) | 1.16 (1.14 - 1.18) |
| **Race/Ethnicity (Ref: Non-Hispanic White)** |  |  |
| Hispanic | 0.94 (0.92 - 0.97) | 0.89 (0.85 - 0.92) |
| Non-Hispanic Black | 0.83 (0.81 - 0.84) | 0.81 (0.78 - 0.83) |
| Non-Hispanic Asian | 0.86 (0.84 - 0.88) | 0.87 (0.85 - 0.89) |
| Non-Hispanic Other | 0.84 (0.82 - 0.86) | 0.84 (0.81 - 0.86) |
| **Limited English Proficiency** | 0.71 (0.69 - 0.72) | 0.70 (0.68 - 0.72) |
| **Medicaid Insurance** | 1.06 (1.05 - 1.08) | 1.02 (1 - 1.05) |
| **Patient Portal Inactivated** | 0.67 (0.66 - 0.68) | 0.61 (0.6 - 0.62) |
| **Visit Type (Ref: Primary Care)** |  |  |
| Behavioral Health | 10.88 (8.78 - 13.48) | 5.53 (4.29 - 7.13) |
| Specialty | 2.26 (2.09 - 2.45) | 2.36 (2.14 - 2.6) |
| **Post Social Restriction** | 0.39 (0.38 - 0.39) | 0.40 (0.39 - 0.4) |
| **Site-Specific Fixed Effect** |  |  |
| Facility 1 | 0.36 (0.25 - 0.53) | - |
| Facility 2 | 0.32 (0.27 - 0.37) | - |
| Facility 3 | 0.10 (0.06 - 0.17) | - |
| Facility 4 | 0.61 (0.53 - 0.7) | - |
| Facility 5 | 0.25 (0.11 - 0.56) | - |
| Facility 6 | 0.19 (0.13 - 0.27) | - |
| Facility 7 | 0.20 (0.14 - 0.28) | - |
| Facility 8 | 0.02 (0.01 - 0.03) | - |
