# Supplementary material for: A privacy-preserving and computation-efficient federated algorithm for generalized linear mixed models to analyze correlated electronic health records data
Source: PLoS One. 2023 Jan 17;18(1):e0280192. doi: 10.1371/journal.pone.0280192 (PMC9844867; doi:10.1371/journal.pone.0280192)
Supplement: S4 Table — We demonstrated variance-component parameter estimates obtained separately for each site to evaluate our assumption of equal variance-component parameters across sites. For sites with a relatively small number of records, the estimates were obtained by fitting the GLMM locally. For sites with a large number of records, the estimates were obtained by a meta-analysis aggregating the GLMM estimates from split data batches. The variance- component parameter estimates for both patient- and physician-level random effects are similar across the sites, supporting our model specification with homogenous variance-component parameters. (DOCX) [file pone.0280192.s006.docx]

| **Facility** | **Variance-Component Parameter Estimate for Patient-level Random Effects** | **Variance-Component Parameter Estimate for Physician-level Random Effects** |
| --- | --- | --- |
| Facility 1 | 0.73 | 2.52 |
| Facility 2 | 0.86 | 2.11 |
| Facility 3 | 1.07 | 2.25 |
| Facility 4 | 0.91 | 2.86 |
| Facility 5 | 0.90 | 2.52 |
| Facility 6 | 0.74 | 1.87 |
| Facility 7 | 0.80 | 2.83 |
| Facility 8 | 1.11 | 1.88 |
